# Gait Recovery After Total Hip Arthroplasty with Subtrochanteric Osteotomy in Highly Dislocated Hips: A Retrospective Single-Center Cohort Study

**DOI:** 10.3390/jcm14207446

**Published:** 2025-10-21

**Authors:** Chan-Jin Park, Gun-Woo Lee, Chan Young Lee, Kyung-Soon Park

**Affiliations:** 1 Department of Orthopedic Surgery, Chonnam National University Medical School and Hwasun Hospital, Hwasun-gun 58128, Jeollanam-do, Republic of Korea; good6621@naver.com (C.-J.P.); cylee0167@naver.com (C.Y.L.); 2 Department of Orthopedic Surgery, Chonnam National University Medical School and Hospital, Gwang-ju 61469, Republic of Korea; gwleeos@gmail.com

**Keywords:** total hip arthroplasty, high hip dislocation, gait analysis

## Abstract

**Background**: We aimed to analyze various gait parameters before and after THA for patients with a highly dislocated hip to examine gait recovery and whether it is continued. **Methods**: This was a retrospective, single-center study. We enrolled 10 patients with a highly dislocated hip (10 hips) due to developmental dysplasia of the hip (DDH) or sequelae of septic arthritis of the hip (SSH). A spatio-temporal gait analysis was performed before THA with subtrochanteric osteotomy and one year after surgery for all patients, and 5 of them had a complete follow-up gait analysis at five years postoperatively. Demographics, clinical outcome, and radiological data were collected. **Results**: At one year postoperatively, the terminal double support (TDS) increased from 8.6% (4.3–12.6) to 11.3% (5.8–14.0) of the gait cycle (*p* = 0.02). The vertical ground reaction force (vGRF) increased from 0.96 N/BW (0.69–1.30) to 1.11 N/BW (0.95–1.31) for the first peak (*p* = 0.045) and from 0.87 N/BW (0.59–1.12) to 1.10 N/BW (1.00–1.30) for the second peak (*p* = 0.001). However, there was no improvement in any gait parameters at five years postoperatively compared to one year postoperatively. The mean HHS was 57.2 (43–67) before surgery and 79.6 (61–88) at the last follow-up (*p* = 0.001). The preoperative leg length discrepancy (LLD), which was 43.6 mm (18.2–71.6), and improved to 9.8 mm (2.1–22.1) after surgery. **Conclusions**: Improvements in stance-phase stability (TDS) and vertical ground reaction forces (vGRF) enhanced gait after THA in patients with highly dislocated hips; however, these gains were only observed until 1 year postoperatively, with no further improvement thereafter. Notably, the magnitude of improvement in TDS and vGRF may exceed that typically reported after THA for primary osteoarthritis.

## 1. Introduction

Total hip arthroplasty (THA) is performed to relieve pain and restore hip function in patients with various hip joint diseases, which is known as the operation of the century [1]. Over the past few decades, various surgical techniques and instruments for THA have been developed, showing promising outcomes. However, THA for a highly dislocated hip is still technically challenging. Most highly dislocated hips are caused by developmental dysplasia of the hip (DDH) or sequelae of septic arthritis of the hip (SSH). Although the surgical technique is rather complicated, THA remains the gold standard of treatment for alleviating pain and restoring joint function in adult patients with a highly dislocated hip [2,3,4,5,6,7].

Several studies have reported the functional results of THA for a highly dislocated hip using clinical outcome measures such as the Western Ontario and McMaster Universities Arthritis Index (WOMAC), Harris Hip Score (HHS), and Modified Merle d’Aubigne-Postel (MAP) score [2,3,4,5,6].

However, these scores have significant limitations, including potential ceiling effects, reliance on patient-subjective reporting, and limited sensitivity in detecting biomechanical changes [8,9].

Recently, gait analysis has been conducted in several studies to quantitatively measure functional improvement after THA [10,11]. However, previous studies have shown that gait function does not fully return to normal even after THA. For example, Bennett et al. reported persistent gait asymmetry in THA patients [12], and Beaulieu et al. found reduced hip extension compared with healthy controls [13]. In addition, other studies compared differences in gait recovery according to surgical approaches or prosthesis types [14,15,16]. Importantly, these findings were primarily observed in patients with normal hip anatomy, such as those with osteonecrosis of the femoral head or primary osteoarthritis. In contrast, patients with highly dislocated hips often require subtrochanteric osteotomy and have distinct anatomical abnormalities, which may result in different gait recovery patterns compared to conventional THA.

To the best of our knowledge, no study has conducted both preoperative and postoperative gait analyses in patients with a highly dislocated hip. We specifically focused on spatiotemporal gait metrics and vertical ground reaction forces (vGRF) because these parameters provide clinically meaningful information: terminal double support (TDS) reflects stability and load acceptance in the stance phase, and vGRF peaks represent the ability of the operated limb to bear weight and generate propulsion [17,18]. Therefore, improvements in TDS and vGRF are directly linked to functional recovery after THA. Thus, this study aimed to investigate changes in spatiotemporal gait parameters and vertical ground reaction forces (vGRF) before and after THA in this population. We hypothesized that spatiotemporal gait parameters and vertical ground reaction force (vGRF) peaks would significantly improve at 1 year postoperatively and continue to show further improvement at 5 years postoperatively, reflecting progressive adaptation and strengthening after THA.

## 2. Materials and Methods

**Figure 1:**
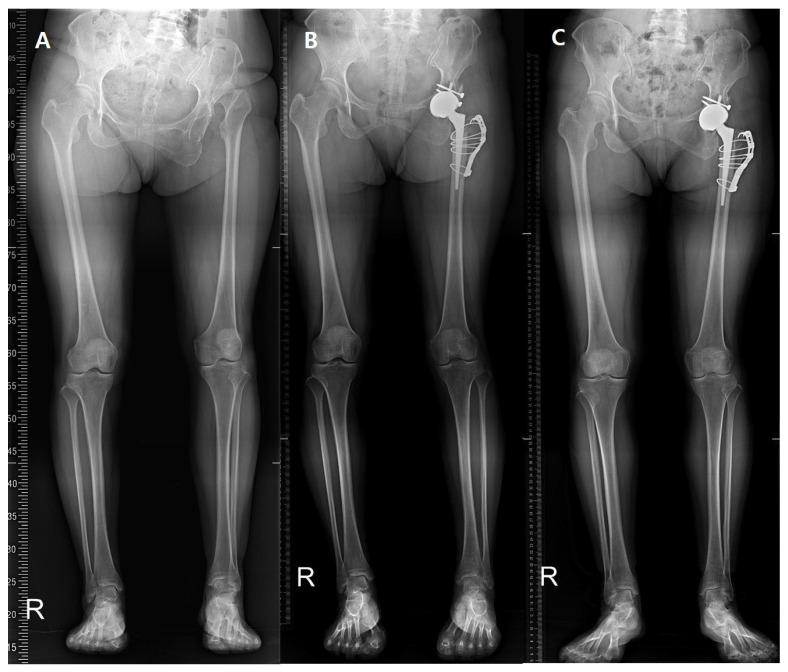
(**A**) Preoperative teleroentgenogram of a 50-year-old female with high hip dislocation due to DDH. (**B**) Postoperative 1-year and (**C**) postoperative 5-year X-rays.

Our Institutional Review Board has approved this study. This was a retrospective, single-center clinical study conducted from November 2012 to November 2013. A total of 18 patients with a highly dislocated hip (total of 15 hips) due to sequelae of SSH or DDH underwent THA with modified subtrochanteric osteotomy [19] (Figure 1). A single hip surgeon performed all THA procedures.

The inclusion criteria were: (1) patients with a highly dislocated hip (Crowe type IV DDH or Hartofilakidis type III SSH); (2) who underwent THA with subtrochanteric osteotomy at our institution; and (3) available pre- and postoperative gait analysis data. The exclusion criteria were: (1) osteotomy-related complications such as nonunion or infection; (2) missing postoperative gait test at 12 months; (3) severe medical comorbidities preventing gait testing; and (4) inability to walk independently before surgery. Of the 15 hips in the study group, we excluded two because of osteotomy-related complications, and three patients missed the postoperative gait test at 12 months. Finally, 10 patients (six females and four males; mean age, 48.9 years; 28–66) were included in this study. The mean follow-up duration was 7.1 years (3–10). There were five patients with Crowe type IV DDH sequelae and five patients with Hartofilakidis type III SSH [20,21]. The patients’ demographic characteristics are summarized in Table 1.

**Figure 2:**
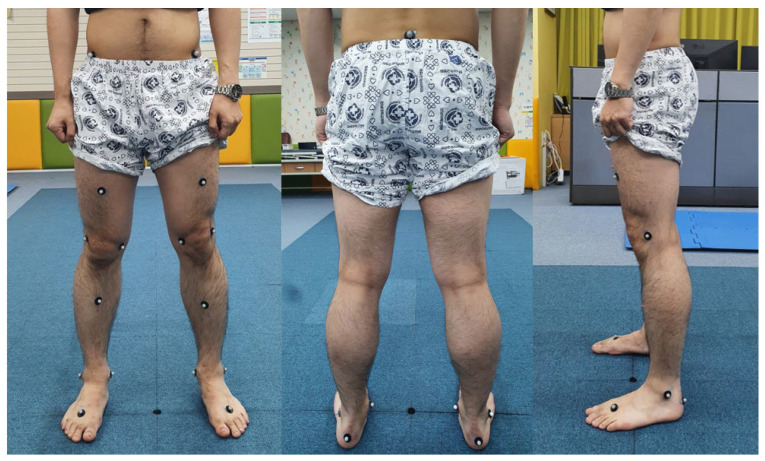
Placement of reflective markers with the Helen–Hayes marker set. A total of 19 markers were placed on each subject.

A spatio-temporal gait analysis was performed before surgery and one year after surgery for all 10 patients, and five of them underwent follow-up gait analysis more than five years after surgery. Of the remaining patients, three were lost to follow-up, and 2 declined the test due to the cost of the examination. The data were subsequently reviewed and analyzed retrospectively. All patients were followed up at 3, 6, and 12 months postoperatively and annually thereafter. In a gait laboratory, patients walked on a 7 m walking path at a self-selected, comfortable speed without external speed control. A total of 19 sphere-shaped reflective markers (Helen–Hayes marker set) were attached to bony landmarks and body segments of the patients to track body movement. Walking speed and cadence were not included as covariates in the statistical analysis, as we aimed to assess gait recovery under natural, self-selected walking conditions rather than under standardized speed. Motion data were recorded by a three-dimensional (3D) high-speed motion capture system (Eight Eagle^®^ digital cameras; Motion Analysis Corporation, Santa Rosa, CA, USA). Additionally, two force plates (Kistler Instrumente AG, Winterthur, Switzerland) were installed on the floor, centered on the walking path, to collect kinetic data using the inverse dynamics method. EVaRT (EVa Real-Time, Version 5.0.5; Motion Analysis Corporation, Santa Rosa, CA, USA) and OrthoTrak (Version 6.6.1; Motion Analysis Corporation, Santa Rosa, CA, USA) were used to analyze the data (Figure 2). Each patient performed three valid walking trials, and the mean values were used for the analysis. Marker trajectories and force data were filtered using a Butterworth low-pass filter with a cutoff frequency of 6 Hz. A single experienced examiner performed all gait analyses following a standardized laboratory protocol to minimize measurement variability. Although intra- and inter-rater reliability were not separately assessed in this study, our gait laboratory has established standardized procedures that ensure consistent marker placement and data processing.

The gait pattern was analyzed using several parameters, including cadence, speed, stride length, step length, step time, initial double support (IDS), terminal double support (TDS), stance phase, and swing phase. In addition, the dynamic range of motion (ROM) of the hip joint and ground-reaction forces (GRFs) were recorded. Dynamic ROM was defined as the peak-to-peak angular excursion (maximum minus minimum angle) of the hip joint during a gait cycle, averaged across the sagittal, frontal, and transverse planes. Time-normalized hip joint angle curves were not available in this retrospective dataset. All data collected by repetitive walking were reduced to mean values by the software.

Clinical (Harris hip scores, HHS) and radiographic evaluations were performed at each visit. Leg length discrepancy (LLD) changes before and after total hip arthroplasty (THA) were measured [19,22].

All statistical analyses were performed using IBM SPSS Statistics for Windows version 25.0 (IBM Corp., Armonk, NY, USA). Normality was tested using the Shapiro–Wilk test, and most variables satisfied the assumption (*p* > 0.05), except for single support, which showed a significant deviation from normality (*p* = 0.011). Therefore, preoperative and postoperative data were primarily compared using paired *t*-tests, and additional Wilcoxon signed-rank tests for this variable yielded consistent results, confirming that the statistical inferences were unchanged. Given the exploratory nature of this study and the small sample size, no formal adjustment for multiple comparisons was applied. A *p* value of less than 0.05 was considered statistically significant.

## 3. Results

All data were compared between the preoperative and postoperative states for the affected limb. Specifically, gait parameters, dynamic range of motion (ROM), and peak forces were compared based on the mean value. At 1 year postoperatively, cadence decreased from 114.7 to 112.6 steps/min (*p* = 0.462), walking speed decreased from 103.9 to 96.4 cm/s (*p* = 0.277), and step length decreased from 52.4 to 50.2 cm (*p* = 0.458). These changes were not statistically significant. On the other hand, compared with the preoperative gait pattern, parameters such as single support, IDS, TDS, and stance phase were increased. In particular, TDS increased from 15.2% to 18.8% of the gait cycle (*p* = 0.020), and the stance phase increased from 58.7% to 62.5% (*p* = 0.001). As the stance phase was extended, the swing phase was reduced from 41.3% to 37.5% (*p* = 0.001) (Table 2).

All patients showed a significant increase in the vertical ground reaction force (vGRF) one year postoperatively. The first peak force (Fz1) increased from 0.96 (0.69–1.30) to 1.11 (0.95–1.31), with a mean difference of +0.186 (95% CI: 0.098–0.273), Cohen’s dz = 1.51 (*p* = 0.045). The second peak force (Fz2) increased from 0.87 (0.59–1.12) to 1.10 (1.00–1.30), with a mean difference of +0.234 (95% CI: 0.107–0.361), Cohen’s dz = 1.32 (*p* = 0.001). At five years postoperation, only five patients were available for gait analysis. In this small subgroup, no further improvement in gait parameters was observed compared to the one-year results. These findings should be regarded as descriptive only due to the limited sample size (Table 2).

For dynamic ROM, the sagittal plane motion was increased, and the transverse and coronal plane motion was decreased. All of the changes were not significantly different between the preoperative and one- and five-year follow-ups.

The vGRF of the contralateral, unaffected hip increased one year postoperatively. The second peak force improved from 0.97 (0.77–1.25) to 1.20 (1.04–1.43), with a mean difference of +0.230 (95% CI: 0.050–0.410), Cohen’s dz = 0.95 (*p* = 0.017) (Table 3). All vGRF values were normalized to Newton/body weight (N/BW) (Table 3).

Clinically, the mean HHS was 57.2 (43–67) before surgery and 79.6 (61–88) at the last follow-up (*p* = 0.001). In radiographic assessment, the patients had a postoperative LLD of 9.8 mm (2.1 to 22.1) at the last follow-up; however, it was significantly improved compared with the preoperative LLD of 43.6 mm (18.2 to 71.6) (*p* = 0.001) (Table 4).

## 4. Discussion

With recent advancements in diagnostic technology, the early diagnosis of DDH and SSH has become possible. However, the late diagnosis of DDH is still prevalent and has not shown a decline [23], and diagnosing septic arthritis of the hip in neonates remains challenging due to the lack of signs and symptoms [24]. Additionally, the incidence of adult-onset septic arthritis of the hip is also on the rise due to the increasing elderly population, the growing number of diabetic patients, the increasing number of immunocompromised individuals, and the prevalence of Methicillin-resistant Staphylococcus aureus [25,26]. A highly dislocated hip, which can occur in the terminal stage of patients with hip joint diseases, results in hip instability with pain, LLD, low back pain, and knee pain. These complications significantly disrupt the static balance of patients’ bodies. THA for a highly dislocated hip is technically challenging due to a dysplastic acetabulum with deficient bone stock, the small diameter of the femoral canal, abnormal anteversion, contracture of the soft tissue surrounding the hip joint, severe flexion deformities, joint stiffness, and limb length discrepancy [27,28]. Nevertheless, THA has shown excellent clinical outcomes as a treatment method for a highly dislocated hip [2,3,4,5,6].

Patients with a highly dislocated hip who underwent THA have shown improved quality of life owing to better joint stability and biomechanics. After THA was successfully performed, these patients can achieve functional recovery and pain relief. However, it was not easy to quantify these subjective data as mentioned above. Nevertheless, laboratory gait evaluation could allow the quantitative identification of functional improvement after THA [10]. Various studies have examined gait changes in patients undergoing THA. Most of these studies involved patients with primary osteoarthritis or osteonecrosis of the femoral head and showed functional improvements, but they still reported persistent gait deficits after surgery, including reduced hip ROM, persistent muscle weakness, and decreased hip abductor moments [11]. However, no previous study has specifically examined changes in gait parameters in patients with a highly dislocated hip. Our study, therefore, provides novel insights into gait recovery in this unique and more challenging patient population.

By conducting a preoperative gait laboratory assessment, we attempted to evaluate not only the differences between unaffected and affected hips but also the changes between the preoperative and postoperative states. Before the postoperative gait laboratory assessment, we expected considerable improvements in all gait parameters. However, the patients showed a decrease rather than an increase in some parameters with residual gait impairment. In our study, all patients showed an improvement in the stance phase. The stance phase consists of the IDS, single support, and TDS. Specifically, the TDS of all patients was significantly increased compared with that in the preoperative state.

Although THA is an effective treatment method for patients with a highly dislocated hip, the underlying mechanism remains unclear. Based on our gait analysis, weight-bearing may be effectively improved by performing THA for patients whose hip joint loading was not properly distributed to the affected lower extremity due to the false acetabulum or pseudo-joint before surgery, which leads to an increase in the TDS in the stance phase. As the stance phase is increased, the swing phase is relatively decreased on the affected limb. These improvements may suggest a reduction in gait asymmetry and antalgic features. However, this interpretation remains indirect, as our study did not include EMG or detailed kinematic analyses to confirm compensatory strategies. Further studies incorporating these methods are needed to validate the underlying mechanisms.

**Figure 3:**
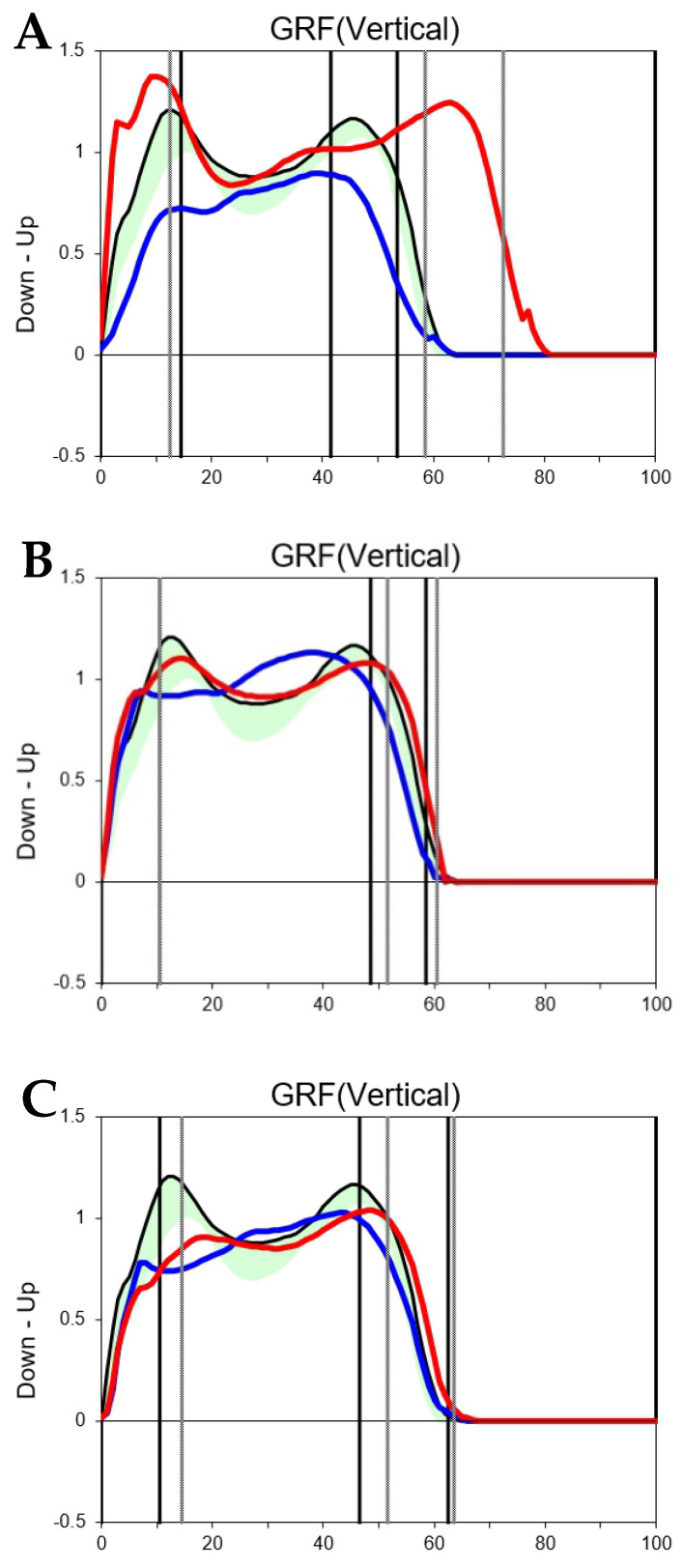
vGRF curves of a patient showing in Figure 1. The red line indicates the unaffected limb, and the blue line indicates the affected limb. The preoperative vGRF curve (**A**) shows a decrease in the Fz1 (1st peak) and Fz2 (2nd peak) values of the affected limb compared with the unaffected limb. The vGRF curves at one year (**B**) and five years (**C**) after surgery show that the curves of the affected and unaffected limbs were similar due to an increase in the Fz1 and Fz2 values of the affected limb.

The significant increase in the first and second peak forces of the vGRF in all patients also suggests that proper hip joint loading in the affected lower extremity may contribute to symptomatic improvement. The vGRF consists of two peaks: the first peak is a passive response (weight acceptance) occurring at heel strike, and the second peak is an active response generated during push-off. The magnitude of these peaks affects the load on the joints and muscles of the lower limb [29,30,31]. McCrory et al. reported that in healthy adults, the first and second vGRF peaks averaged 1.05 and 1.02 N/BW, respectively. In contrast, in patients after conventional THA, these peaks were slightly lower (1.02 and 1.00 N/BW) [32]. In contrast, our postoperative values (1.11 and 1.10 N/BW) were even higher than those reported for healthy adults. This discrepancy may reflect differences in patient populations: our cohort included patients with highly dislocated hips who had markedly reduced preoperative loading due to pseudo-joint articulation. It thus demonstrated a greater relative improvement after anatomical reconstruction. Therefore, painless locomotion with the reconstructed hip joint may have allowed stronger and longer heel strike and push-off, resulting in the significant increase in the first and second peaks observed in this study (Figure 3).

An improvement in the vGRF was also observed in the contralateral, unaffected hip after surgery, which may reflect an overall balancing of load distribution resulting from the functional recovery of the affected hip.

By five years postoperatively, the vGRF had decreased compared to its level at one year, which was inconsistent with our initial hypothesis that gait function would continue to improve through progressive adaptation after THA. This reduction is likely multifactorial. In addition to possible adaptation and aging, other factors such as implant wear, progressive muscle weakness, or comorbidities may have contributed. However, these long-term findings should be interpreted with caution because of the small number of patients available at five years.

Our study has several limitations. First, the cohort size was relatively small, and no a priori power analysis was performed because the study was retrospective and included all eligible patients during the study period. Post hoc power analysis (two-tailed α = 0.05; *n* = 10) showed that the paired-sample effect sizes and achieved power were as follows: TDS (Cohen’s dz = 0.89, power = 0.71), vGRF 1st peak (Cohen’s dz = 1.51, power = 0.998), and vGRF 2nd peak (Cohen’s dz = 1.32, power = 0.99). However, smaller differences in spatiotemporal parameters, such as cadence or step length, may have been underpowered. Additionally, gait analysis was performed at five years after surgery for only 5 of the 10 patients. However, the baseline clinical and gait characteristics of patients lost to follow-up or who declined testing were similar to those of patients who completed the 5-year evaluation, suggesting that loss to follow-up is unlikely to have systematically biased the long-term results. Thus, the 5-year findings should be regarded as descriptive only, and their statistical validity is limited. Second, the gait speed was not normalized across patients, which may have influenced the spatiotemporal and kinetic outcomes. Because all trials were performed at self-selected speeds, these findings should be interpreted with this limitation in mind. No covariate adjustment for walking speed or cadence was performed, as the study intentionally evaluated gait recovery during natural, self-selected walking to reflect real-world functional performance. Third, we reported only the averaged dynamic ROM values rather than full-time normalized angular curves. This limits the ability to capture detailed kinematic deviations throughout the gait cycle. Finally, EMG and detailed kinematic data were not collected, and compensatory strategies could not be directly assessed. In addition, intra- and inter-rater reliability were not specifically evaluated; however, all gait analyses were conducted by a single, experienced examiner using a standardized laboratory protocol to minimize potential measurement variability. Nevertheless, to the best of our knowledge, this was the first study to evaluate gait characteristics before and after THA in patients with a highly dislocated hip and to compare the affected and unaffected sides within the same individuals.

## 5. Conclusions

Although THA is known to be a highly effective treatment method for patients with a highly dislocated hip, the exact mechanism has not been fully elucidated by gait analysis. In our study, we found that improvements in the stance phase, specifically TDS, and increases in vGRF contributed to better gait ability in this population. However, these improvements were observed only until one year postoperatively, with no further gains thereafter. Notably, the magnitude of improvement in TDS and vGRF may be greater than that typically reported after THA performed for primary osteoarthritis, suggesting that patients with highly dislocated hips may experience more pronounced functional gains once proper hip joint loading is restored.

## Figures and Tables

**Table 1:** Patient Demographics.

Parameters	
Age (years)	48.9 (28 to 66)
Female/male	6/4
Body mass index (kg/m^2^)	29.8 (24.5 to 38.2)
Preoperative status	
DDH (Crowe type)	5 (IV)
SSH (Hartofilakidis type)	5 (III)
Follow-up (years)	7.1 (3 to 10)

The values are given as an average and range. DDH, developmental dysplasia of the hip; SSH, sequelae of septic arthritis of the hip.

**Table 2:** Comparison of Gait Parameters of Affected Hip Pre- and Post-Operation.

	Pre-Operative (N = 10)	Post-Operative 1-Year (N = 10)	Post-Operative 5-Year (N = 5)	*p* Value (Pre- vs. 1-Year)	*p* Value (Pre- vs. 5-Year)	*p* Value (1- vs. 5-Year)
**Spatio-temporal**						
**Cadence (steps/min)**	114.7 (100.1–128.7)	112.6 (93.8–127.3)	110.6 (93.4–117.8)	0.462	0.742	0.852
**Speed (cm/s)**	103.9 (82.4–139.1)	96.4 (68.6–126.5)	98.8 (88.6–109.8)	0.277	0.835	0.765
**Stride length (cm)**	108.5 (83.7–132.6)	102.3 (88.0–119.0)	107.9 (93.7–116.6)	0.277	0.640	0.752
**Step length (cm)**	52.4 (37.4–69.4)	50.2 (42.0–59.5)	53.3 (43.6–60.1)	0.458	0.437	0.956
**Step time (s)**	0.53 (0.47–0.60)	0.54 (0.47–0.64)	0.55 (0.51–0.64)	0.541	0.771	0.764
**Single support (% cycle)**	35.6 (27.2–40.6)	37.2 (30.2–39.9)	37.4 (35.8–39.8)	0.281	0.262	0.922
**IDS (% cycle)**	10.1 (6.2–14.7)	11.6 (8.1–14.2)	10.7 (8.2–12.3)	0.171	0.988	0.654
**TDS (% cycle)**	8.6 (4.3–12.6)	11.3 (5.8–14.0)	13.2 (11.0–15.7)	0.020	0.024	0.953
**Stance phase (% cycle)**	54.4 (49.5–59.7)	60.1 (56.5–67.4)	61.4 (59.1–62.9)	0.001	0.003	0.945
**Swing phase (% cycle)**	45.6 (40.3–50.5)	39.9 (32.6–43.5)	38.6 (37.1–40.9)	0.001	0.003	0.965
**Vertical GRF**						
**1st peak force (** **N/BW)**	0.96 (0.69–1.30)	1.11 (0.95–1.31)	0.91 (0.78–0.99)	0.045	0.240	0.856
**2nd peak force (** **N/BW)**	0.87 (0.59–1.12)	1.10 (1.00–1.30)	1.01 (0.97–1.03)	0.001	0.307	0.621
**Dynamic ROM**						
**Sagittal**	25.8 (11.9–50.4)	30.8 (19.3–53.3)	37.0 (30.1–53.8)	0.257	0.051	0.652
**Transverse**	13.6 (5.5–24.2)	11.3 (5.7–16.8)	11.4 (8.1–16.6)	0.286	0.099	0.564
**Coronal**	10.0 (5.6–16.3)	7.4 (3.4 –14.3)	8.2 (5.2–11.0)	0.115	0.671	0.620

The values are given as an average and range. IDS, initial double support; TDS, terminal double support; GRF, ground reaction force; BW, body weight; ROM, range of motion.

**Table 3:** Comparison of Vertical GRF Changes in the Unaffected Hip Pre- and Post-Operation.

	Pre-Operative (N = 10)	Post-Operative 1-Year (N = 10)	Post-Operative 5-Year (N = 5)	*p* Value (Pre- vs. 1-Year)	*p* Value (Pre- vs. 5-Year)	*p* Value (1- vs. 5-Year)
1st peak force (N/BW)	1.05 (0.73–1.39)	1.23 (1.05–1.70)	0.96 (0.91–1.03)	0.102	0.120	0.850
2nd peak force (N/BW)	0.97 (0.77–1.25)	1.20 (1.04–1.43)	1.03 (0.98–1.09)	0.017	0.652	0.782

The values are given in average value and range. GRF, ground reaction force; BW, body weight.

**Table 4:** Comparison of clinical and radiographic changes between pre-operation and last follow-up.

	Pre-Operative	Post-Operative	*p* Value
HHS	57.2 (43–67)	79.6 (61–88)	0.001
LLD (mm)	43.6 (18.2–71.6)	9.8 (2.1–22.1)	0.001

The values are given in average value and range. HHS, Harris Hip Score; LLD, leg length discrepancy.

## Data Availability

The datasets generated and/or analyzed during the current study are not publicly available due to institutional privacy policies and patient confidentiality but are available from the corresponding author upon reasonable request.
